# Supplementary material for: Multi-model methods for structural analysis of China’s green economy network based on input-output method
Source: PLoS One. 2024 Sep 5;19(9):e0309916. doi: 10.1371/journal.pone.0309916 (PMC11376532; doi:10.1371/journal.pone.0309916)
Supplement: Attachment — Submitted filename: Response to Reviewers.docx [file pone.0309916.s002.docx]

# Detailed response to reviewer's comments

Manuscript numbers: **PONE-D-23-30302**

Title: " **Multi-Model Methods for Structural Analysis of China's Green Economy Network Based on Input-Output Method**"

Correspondence Author: **Shaoling Ding**

Dear editors and reviewers:

Thanks very much for your time to review this manuscript. All your comments are both professional and valuable, which are of great significance improving our scientific research value and the quality of essay writing. We have considered these comments carefully and tried our best to address every one of them. We hope the manuscript after careful revisions meet your high standards. The authors welcome further constructive comments if any.

We have studied comments carefully and have made correction which we hope meet with approval. The summary of corrections and the responses to the editor's and reviewer’s comments are listed in the Revision Report.

Note: In the response letter, **all the editor’s and reviewer’s comments are shown in blue**, and **all the responses are shown in black**, and **all the changes are shown in red**. Also, in the revised paper, **all the changes and additions are highlighted in yellow.**

Thank you and best regards.

Yours sincerely,

Shaoling Ding

# Revision Report.

First of all, we are grateful to the editor and reviewers for their comments. These comments are all valuable and helpful for revising and improving our manuscript, as well as the essential guiding significance to our research. We have studied the comments carefully and have made corrections, which we hope meet with approval. The summary of corrections and the responses to the editor’s and the reviewer’s comments are listed below.

**Summary of the revision:**

**Section 1(Introduction):** We have added some specific figures and information about China’s green industry and presented them as pictures. In addition, we have re-described the background description and the methodology used in the introduction of the whole text. Furthermore, we produced a Research Roadmap figure for the full text.

**Section 2 & Section 3:** We modify the expression of some technical terminologies. Moreover, we have abbreviated the method that appears several times in the article.

**Section 4(Experiments)：**We have recharacterized the experimentally demonstrated results. In addition, we added the findings and literature of previous research on the green economy and green industry to support the article’s credibility.

**Section 5(Conclusion)：**We have briefly re-described this paper’s research methodology and themes at the conclusion. Moreover, we have divided the Conclusions and Recommendations into three points respectively. And at the end, we add the deficiencies of this study and future research directions.

In addition, the manuscript has been carefully revised considering the language and grammar problems, including the sentences that are convoluted and hard to follow.

# Response to Editor and Reviewers

**Editor #:**

**Comment 1:** When submitting your revision, we need you to address these additional requirements. Please ensure that your manuscript meets PLOS ONE’s style requirements, including those for file naming.

**Response 1:** Thank you for your valuable comments on the style of the paper. We have checked the manuscript throughout and also revised manuscript according to *PLOS ONE* in the revised manuscript.

**Comment 2:** Did you know that depositing data in a repository is associated with up to a 25% citation advantage? If you’ve not already done so, consider depositing your raw data in a repository to ensure your work is read, appreciated and cited by the largest possible audience. You’ll also earn an Accessible Data icon on your published paper if you deposit your data in any participating repository.

**Response 2:** Thanks for your notifications. We already know that putting data into repositories makes it read, appreciated, and cited by a wider audience. We are honored that our data is available to a broader audience, so we have packaged the data in an attachment named **S1 File. sample data**.

**Comment 3:** We note that you have provided funding information that is not currently declared in your Funding Statement. However, funding information should not appear in the Acknowledgments section or other areas of your manuscript. We will only publish funding information present in the Funding Statement section of the online submission form. Please remove any funding-related text from the manuscript and let us know how you would like to update your Funding Statement.

**Response 3:** Thanks for your attention, which is highly appreciated. We have removed the Acknowledgments Section of the manuscript regarding the funding information and revised the Acknowledgments. We would appreciate your help in revising the funding information in the online submission. The revisions are listed below:

Funding Statement: The authors acknowledge that this work was supported by the Guangxi Colleges and Universities Key Laboratory of Applied Statistics, and College of Science, Guilin University of Technology. This research is funded by the National Natural Science Foundation of China，grant number 72263004; the Natural Science Foundation of Guangxi Province，grant number 2022JJB180025; and the Guilin University of Technology Research Initiation Fund Project, grant number GUTQDJJ6613056. The funders had no role in study design, data collection and analysis, decision to publish, or preparation of the manuscript.

**Reviewers #:**

**Reviewer 1:**

**Comment 1**: Please present some specific figures or values of the green industries in China for the analyzed period into Introduction.

**Response 1:** Thank you very much for your valuable suggestions in the Introduction of this paper. There is yet to be a clear delineation of the green economy and industry globally, so China has not yet published precise official data on the development of the green economy. Therefore, we refer to the report on the development of green economy-related industries and the data on green development therein. We have added information and figures about the status of China’s Green Development and its related indicators in the Introduction Section, as shown in Fig 1, so readers can understand them intuitively. We have briefly added this discussion in the first paragraph of Section 1:“As shown in Fig 1 (a), China’s Green Development Index has improved from 53.01 in 2016 to 70.12 in 2020, an increase of 32.23%. In addition, the secondary indicators of the Green Development Index all have significant improvements of different degrees. The development of green economy proposes solutions to China’s environmental problems and becomes an important driving force for China’s economic development mode and industrial transformation and upgrading.”

Fig 1. China’s Green Economy Indicators Index

**Comment 2**: Correlate your results with previous studies on this topic in China or other countries.

**Response 2**: We sincerely appreciate the valuable comments. By reading the literature on the direction of the Green Economy, we have found several literatures that can be linked to this article. We added this work to the Section 4 of the article:

First,“This is because after 2016, China guided the development of low-carbon industries, actively explored the transformation of traditional industries, and promoted the modernization of industrial chains with new technologies[1].In addition, according to data from China’s Ministry of Commerce, the proportion of foreign investment in high-tech industries has increased from 14.1% in 2012 to 36.1% in 2022, an increase of more than one times. During this period, the structure of China’s actual use of foreign investment was gradually upgraded, and the amount and level of foreign direct investment increased significantly. This move can promote economic cooperation, technological innovation, and resource sharing between regions and countries. It can help China realize the modernization and upgrading of the industrial chain as soon as possible[2].”

Second,“China’s central industries are mainly distributed in the secondary industry, because China is the world’s largest manufacturing country, and the industrial output value occupies an important position in the national economy. These industries form a high degree of resource dependence on other industries[3].”

Third,“With the growth of China’s economy and the upgrading of industrial structure, the clustering role played by the tertiary industry has become increasingly prominent[4].”

Fourth,“Similar to other scholars’ research, C4- electricity, heat production and supply and A1-agricultural products ranked high in terms of industrial influence, especially C4-electricity, heat production and supply ranked in the top three[3].”

Fifth, “On the other hand, China has achieved remarkable results in ecological and environmental governance, but due to the differences in high-quality development between regions, underdeveloped regions and some regions that have fallen into the dilemma of development and transformation are lagging in the adjustment of energy structure and the application of energy-saving and emission reduction technologies[5].”

1.	Yu ZZ, Lei G, Jian Z. How national policies facilitate low carbon city development: A China study. Journal of Cleaner Production. 2019;234:743-54.

2.	Lu YL, Zhang YQ, Cao XH, Wang CC, Wang YC, Zhang M, et al. Forty years of reform and opening up: China’s progress toward a sustainable path. Science advances. 2019;5(8):eaau9413.

3.	Rui W. Ecological network analysis of China’s energy-related input from the supply side. Journal of Cleaner Production. 2020;272:122796.

4.	Lei C, Hu LJ, Fang H. Empirical Examination of Factor Mobility, Industrial Agglomeration and Economic Development. Statistics and Decision. 2021.

5.	Jiang HB, Zou YN, Wei G, Zhen Y, Yu H, Tao L. Annual report on the high quality development of China （2022）2022.

**Comment 3**: Add limitations and directions for further research at the end of Conclusions.

**Response 3:** Thank you very much for your professional and constructive advice. In the process of writing and revising the article, we found the limitations of this paper. Also, we found innovative points from it to provide an idea for future research directions. With reference to your comments, we think it is necessary to add the limitations of this paper and directions for further research. And, we have added this work in the last paragraph of Section 5 and generated the 5.3 Outlook Section:“Although this study effectively identifies the status and role of green industries and green economy in Chinese industries based on the input-output method, there are still some limitations. First, this paper analyzes the dynamic changes of green industries, but due to the data limitation that the IOT is updated every five years, the data years collected are too few to fully show the evolution process of the industrial structure. The volume of indicator data could be expanded in future studies. In addition, this study used a variety of comprehensive methods such as MST method, LCC algorithm and threshold screening to effectively identify industrial chains and key green industries, but did not further analyze the factors influencing such phenomena, which is also the direction of subsequent research. Third, we can moderately increase the research content of the spatial pattern changes and spatiotemporal comparative analysis of the development of China’s green economy by conducting an overall analysis of China’s green industry.”

**Reviewer 2:**

In general, this manuscript is focused on the research of green economy by using the traditional input-output method, which has good and insightful topic selection, significance emphasizing, text writing, method using, expansion studies, and conclusion summarization. Only minor revision is needed to perfect the overall content.

**Response:** We feel great thanks for your professional comments on our paper, which will improve the readability and professionalism of the paper. We have responded point-by-point to your comments.

**Comment 1:** In Introduction, please consider adding some figures & statistical graph that can visualize this research. E.g. the green economy level in different regions in China, or the proper relevant statistical data charts.

**Response 1:** We highly appreciate the constructive suggestions of the reviewer regarding the Introduction. By searching a large amount of literature and materials, we found that Chinese officials have not yet explicitly published detailed data on the development of the green economy and industries. There may be a reason why the green economy and its industries have not yet been clearly defined globally. Therefore, we refer to the report on the development of green economy-related industries and its data on green development. In the "Introduction" section, we have added information and data on the current status of China’s green development and related indicators. We present them with the information in Fig 1 so that readers can visualize them. We have added this discussion according to your comments in the first paragraph of Section 1:“As shown in Fig 1 (a), China’s Green Development Index has improved from 53.01 in 2016 to 70.12 in 2020, an increase of 32.23%. In addition, the secondary indicators of the Green Development Index all have significant improvements of different degrees. The development of green economy proposes solutions to China’s environmental problems and becomes an important driving force for China’s economic development mode and industrial transformation and upgrading.”

Fig 1. China’s Green Economy Indicators Index

**Comment 2:** It would be better to add a research roadmap of the whole paper in the introduction.

**Response 2:** Thank you very much for your suggestions regarding the framework of the paper to further help improve the standardization of the paper. Referring to your suggestion, adding a research roadmap is beneficial to improve the completeness and professionalism of the article. Therefore, we have added the research roadmap of the paper in the Introduction section, as shown in Fig 2.

Fig 2. Research Roadmap

**Comment 3:** In conclusion, please briefly re-describe the research topic and methods in this study.

**Response 3:** Thank you very much for your insightful comments. We re-describe the topic and methods of the paper in the Introduction and Conclusion to highlight the research objectives and application methods of the articles.

We have revised this work in the Section 1:“Given the current importance of the green economy as a development strategy in China, several urgent questions need to be investigated and researched: What is the position of the green economy and its related industries in China’s economic development? What direct and indirect effects exist among these industries? What are the industrial community effects in the green economy industry? Under the current financial foundation, which specific areas of development within the green economy are more conducive to economic growth? Based on the above considerations, while the mentioned research content can provide theoretical guidance and efficiency evaluation of the green economy, it cannot accurately reflect the effect relationship between industries. Therefore, to better analyze the internal industry relationships between the green industry and other industries, this study applies the input-output method based on sectoral data. From a complex network perspective, the industries are treated as network nodes of equal status, with input-output relationships between industries as network edges. Therefore, by transforming the complex influence effect relationship between industries into the data structure of the complex network, the characteristics and development trends of industrial structure exhibited by the green economy are analyzed.” “In the network, what is the position of the green economy and its related industries in China’s economic development? What direct and indirect effects exist among these industries? What are the industrial community effects in the green economy industry? Under the current financial foundation, which specific areas of development within the green economy are more conducive to economic growth? To solve these problems, this paper transforms the complex influence effect relationship between industries based on the input and output data in different periods into the data structure of a complex network. It constructs a dynamic industrial network model to analyze the characteristics and development trends of the industrial linkage structure demonstrated by the green economy. Due to the multidimensional nature of the network structure, we analyze the industry structure through overall metrics and node metrics. The overall characteristics are measured by topological properties such as network density and average shortest path. The MST method is used to find the backbone network in the industry and analyze the correlation shown by each industry node in the network and the community structure based on the network graph. In addition, LCC algorithm is introduced as a complementary indicator of the nodes to analyze the role played by the green economy and its industries in the community. Considering the heterogeneity of nodes, the importance of each indicator is uncertain. Therefore, we use the WT model on the basis of entropy weight method to get the importance ranking of industrial nodes and analyze the position and role of green economy in China’s national economic system.”

Similarly we have revised this work in the Section 5：“With the increasing emphasis on green and sustainable development worldwide, green measures such as sustainability, carbon neutrality, and peak carbon emissions are gradually being implemented. Issues related to economic development, ecological environment protection, and governance have become fundamental strategies and essential driving forces for stimulating the transformation and development of the world economy. Especially in the late emerging countries represented by China, on the road to economic transformation and breakthrough, how to achieve economic growth, efficiently use resources, and protect the environment. For these countries, a series of development challenges remain. Therefore, in order to explore the current development status of China’s green economy, this article synthesizes Chinese green economy and its development status and draws the following main conclusions.” “With the increasing emphasis on green and sustainable development worldwide, green measures such as sustainability, carbon neutrality, and peak carbon emissions are gradually being implemented. Issues related to economic development, ecological environment protection, and governance have become fundamental strategies and essential driving forces for stimulating the transformation and development of the world economy. Therefore, this study is based on four sets of input-output data from China from 2012 to 2020, combined with industrial networks and input-output methods, to construct a dynamic industrial network model to analyze the development status of China’s green industry. We analyze the overall structure of industry through the topological properties and network density of the industrial network. Then, we use the MST method and the LCC algorithm to analyze the associations and cluster effects of the green economy and its industries in the industrial network. The WT model was used to evaluate the position and role of the green economy and its industries in China.”

**Comment 4:** In conclusion, please consider adjusting the conclusions and recommendations into 3 points respectively, and adding research deficiencies and possible future studies in your team.

**Response 4:** We would greatly appreciate the reviewers’ comments on the final part of the paper. We have elaborated the contents of the conclusions and recommendations into 3 points respectively. While writing and revising the article, we found that the article has certain deficiencies. At the same time, we also see the direction of future research from it. Based on your suggestion, we believe that the addition of this section is necessary. Based on your suggestions, we think it is necessary to add the research deficiencies and research directions in the future. And, we have added this work in the last paragraph of Section 5 and generated the 5.3 Outlook Section:“Although this study effectively identifies the status and role of green industries and green economy in Chinese industries based on the input-output method, there are still some limitations. First, this paper analyzes the dynamic changes of green industries, but due to the data limitation that the IOT is updated every five years, the data years collected are too few to fully show the evolution process of the industrial structure. The volume of indicator data could be expanded in future studies. In addition, this study used a variety of comprehensive methods such as MST method, LCC algorithm and threshold screening to effectively identify industrial chains and key green industries, but did not further analyze the factors influencing such phenomena, which is also the direction of subsequent research. Third, we can moderately increase the research content of the spatial pattern changes and spatiotemporal comparative analysis of the development of China’s green economy by conducting an overall analysis of China’s green industry.”

**Comment 5:** Please confirm that all the Figures uploaded are in high resolution.

**Response 5:** Thanks for your helpful comments. In our subsequent checking work, we realized that the Figures we submitted individually had their picture quality compressed in the documentation. Moreover，we have re-processed all the Figures to be precise while having a resolution of 300 dpi.

**Comment 6:** Finally, please carefully re-check the English writing, including the sentence expressions, grammar and technical terminologies.

**Response 6:** Thank you very much for your suggestions on further improving the paper writing. After reading the article carefully, we tried our best to improve the manuscript and made some changes to the manuscript. These changes do not affect the content or framework of the paper. Instead of listing these changes here, we have highlighted them in yellow in the revised paper.
