# Supplementary material for: Multi-model methods for structural analysis of China’s green economy network based on input-output method
Source: PLoS One. 2024 Sep 5;19(9):e0309916. doi: 10.1371/journal.pone.0309916 (PMC11376532; doi:10.1371/journal.pone.0309916)
Supplement: Attachment — Submitted filename: Response to Reviewers.docx [file pone.0309916.s003.docx]

# Detailed response to reviewer's comments

Manuscript numbers: **PONE-D-23-30302R1**

Title: " **Multi-Model Methods for Structural Analysis of China's Green Economy Network Based on Input-Output Method**"

Correspondence Author: **Shaoling Ding**

Dear editors and reviewers:

Thanks very much for your time to review this manuscript. All your comments are both professional and valuable, which are of great significance improving our scientific research value and the quality of essay writing. We have considered these comments carefully and tried our best to address every one of them. We hope the manuscript after careful revisions meet your high standards. The authors welcome further constructive comments if any.

We have studied comments carefully and have made correction which we hope meet with approval. The summary of corrections and the responses to the editor's and reviewer’s comments are listed in the Revision Report.

Note: In the response letter, **all the editor’s and reviewer’s comments are shown in blue**, and **all the responses are shown in black**, and **all the changes are shown in red**. Also, in the revised paper, **all the changes and additions are highlighted in yellow.**

Thank you and best regards.

Yours sincerely,

Shaoling Ding

# Revision Report.

We would like to express our gratitude to the editors and reviewers for their valuable comments, which have been instrumental in revising and improving our manuscript. We have carefully studied the comments and made necessary corrections, and we hope that they will be approved. The summary of corrections and our responses to the editor's and reviewers' comments are outlined below.

**Summary of the revision:**

**Section 5(Conclusion)：**We have revised the recommendations section to make them more actionable and targeted.

In addition, the manuscript was carefully revised taking into account grammatical and formatting issues.

# Response to Editor and Reviewers

**Editor #:**

**Comment 1:** If you would like to make changes to your financial disclosure, please include your updated statement in your cover letter.

**Response:** Thank you very much for your work on this manuscript. We would appreciate your help in revising the funding information in the online submission. The revisions are listed below:

Funding Statement: The authors acknowledge that this work was supported by the School of Mathematics and Statistics, Guilin University of Technology, and the Guangxi Colleges and Universities Key Laboratory of Applied Statistics. This research is funded by the National Natural Science Foundation of China, grant number 72263004; the Guangxi Natural Science Foundation, grant number 2023GXNSFBA026171; and the Guilin University of Technology Research Initiation Fund Project, grant number GUTQDJJ6613056. The funders had no role in study design, data collection and analysis, decision to publish, or preparation of the manuscript.

**Reviewers #:**

**Reviewer 1:**

No further comments. The comments were properly adressed and I believe that he paper can be published in the current form.

**Response:** Thank you very much for your suggestions for this article, and thank you again for your support of our work.

**Reviewer 2:**

After the review & modification works, the review comments have been seriously considered by the author(s). The manuscript R1 now shows sound topic expression, academic writing, logical essay structure, and adequate quantitative methods. Most of the deficiencies have been solved, thereafter, I personally do not have any more comments for the manuscript R1.

Summing up the above, my personal recommendation is ACCEPT. If accepted and in the proofreading stage, please re-check carefully on the manuscript writing, grammar, expressions, and possible omitted interpretations of the research topics, method descriptions and results discussion.

**Response:** We sincerely appreciate the valuable comments. We've corrected the grammar and formatting of the article. Thank you again for your recognition and support of our work.

**Reviewer 3:**

The authors carefully revised the questions raised by the reviewers. The revised manuscript has achieved the expected effect and met the basic requirements of the journal.

**Response:** Thank you for reading this article carefully and giving it a high rating. We are very grateful for your support of our work.

**Reviewer 4:**

This study uses industry networks, the maximum spanning tree (MST) method, the Leiden community clustering (LCC) algorithm, and the Weaver-Thomas (WT) model to analyze the contribution and status of China's green economy in economic development. The data were obtained from the input-output tables published by the National Bureau of Statistics of China in 2012, 2017, 2018 and 2020. The industry network was constructed by internal flow matrix and total consumption coefficient matrix, and topological characterization, MST method to extract backbone network structure, and LCC algorithm for network community analysis were applied. Finally, the WT model is used to determine the importance of industry nodes and analyze their position in China's national economic system. However, the manuscript still has some issues to be improved.

**Response:** We sincerely appreciate your professional comments on our paper, which has significantly enhanced its readability and professionalism. Below, we have addressed each of your comments.

**Comment 1:** Please further explain the theoretical and practical significance of this study.

**Response 1:** Thank you very much for your helpful suggestions. Due to space limitations, abbreviations have been used in this section of the article. In this response, we have provided detailed answers to your questions.

Theoretical significance: Firstly, the topic of the green economy and industrial development has always been a hot topic in academic research. Moreover, the current research on the green economy and industry is relatively small compared with crucial development areas such as the digital economy. Based on the input-output table, this paper provides a research framework for analyzing the relationship between the green economy and its industry from the perspective of complex networks, expands the application of the input-output table (IOT) in the green economy and its industries, and provides new research perspectives and methods for related research. Secondly, the industrial sectors of the green economy and its related industries are clearly defined by subdividing, dismantling, adjusting, and dividing the relevant parts of the original IOT. This enriches the statistical classification system of various industrial sectors of the national economy in the IOT. Thirdly, this research framework takes the correlation between industries and industries as the research content and uses the spanning tree algorithm, community mining, threshold network and other calculation methods. This research framework can analyze the various structural characteristics of specific industries in the actual industrial network, find the connections between different industries, and compare the importance and development trends of different industries.

Practical significance: As the role of the green economy in China's economic system becomes increasingly prominent, its influence is gradually increasing. The analysis based on IOT can help provide policy recommendations for the green economy and its industrial development planning. With the progress of science and technology, industries differ from the previous industries. Many industries and all walks of life can be related and integrated, and the intricate interaction between industries needs to be analyzed using the industrial network model framework. Based on the IOT released by the Chinese government, the industrial network was constructed, and the structural characteristics, importance, development trend and industrial impact of green industries were analyzed. We can identify the core structure of China's green economy and identify the green industries that have the strongest driving effect. In this way, we will strive to promote critical industries to move towards green and environmental protection to guide China's green economic development planning and policy formulation. Therefore, it is not only of great practical significance to deeply explore the characteristics of the green economy and its industries in the network structure but also the key to promoting the transformation of economic development mode, promoting the development of the green economy and vigorously releasing its efficiency.

**Comment 2:** What is the basis for the article's selection of industry network, maximum spanning tree (MST) method, Leiden community clustering (LCC) algorithm and Weaver-Thomas (WT) model? What are the applicability and limitations of these methods?

**Response 2:** Thanks for your helpful comments. Due to space constraints, this section of the article needs to be elaborated. Therefore, we address your doubts in this response. With the advancement of science and technology, industries differ from those of the past. Many industries and all walks of life can be related and integrated, and the traditional analysis methods of econometrics or input-output methods may have limitations in studying this aspect. We aim to examine the complex industrial linkages, convergences, and interactions between green economy sectors and other industries from the perspective of industrial interconnections. This complex inter-industry linkage requires using complex network analysis methods for modelling and metric analysis.

By analyzing the input-output network, we can not only reveal the general relationship law between departments but also better predict and deduce the future scenarios of economic operation. After the industrial network is constructed according to China's IOT (Fig 3), we find that the number of connection edges between industries is large, and the industrial network structure is dense and complex, which makes it challenging to analyze the most significant relationships within the network intuitively. Therefore, we utilized a backbone network extraction algorithm, precisely the maximum spanning tree (MST) method to identify and extract the most critical and closely linked input-output relationships within industries. By discarding less essential or irrelevant connections in the network, we obtained a clear framework of industrial chains and clusters, facilitating detailed analysis from an industrial economic perspective. However, we recognized some limitations in the industrial networks and the maximum spanning tree algorithm. Acquiring data, constructing network structures, and determining which connections to include in the network are critical challenges in industrial network analysis. Similarly, the MST method primarily relies on edge weights to construct the spanning tree without directly considering node attributes or other internal structural information, which may limit its ability to fully capture the network's complexity in some cases.

Fig 3. Industry network timing diagram

From the perspective of industrial networks, relationships between industries vary in density. These differing degrees of connectivity manifest in the network structure, forming clusters of interconnected nodes that reflect as industrial clusters in economic activities. These aggregated groups can be identified using network clustering algorithms, ultimately revealing distinct industrial clusters characterized by closely interconnected relationships. We aim to explore industrial clusters to identify tightly interconnected groups of industries. Therefore, the Leiden community clustering (LCC) algorithm, which is common in the network structure, is optimized based on the traditional network Louvain community clustering algorithm. It can ensure the connectivity between nodes in the community and, at the same time, obtain more stable high-quality partitions. However, it may have limitations when applied to large-scale networks, leading to computational complexity and performance challenges.

The MST algorithm is used in the transparent network to identify the primary radiation industries of the green industry and find the leading industries among the many industries. Since the evaluation of industries needs to be considered from the perspective of inter-industry linkage and comprehensiveness, we use the Weaver-Thomas (WT) model for this purpose. This model improves the defect of determining the number of industries according to the empirical distribution and hypothetical distribution pairs in the traditional algorithm[1]. It also solves the problem of industrial optimization analysis under the condition of multiple indicators.

This method has become essential for countries to evaluate and select industries. The basic principle is to compare an observed distribution with a hypothetical one to construct the closest approximate distribution. Compared with other methods, the model is more targeted in industrial research and can identify the leading industries more scientifically and accurately. Like the LCC algorithm, the WT model requires much time and resources for large-scale data processing and analysis.

**Comment 3:** The selection of parameters and variables involved in the calculations lacks sufficient theoretical support and empirical basis.

**Response 3:** Thank you very much for your insightful comments. In the current global challenging context, China faces both challenges and opportunities. For China, just like the digital economy, the green economy is an effective strategy to tackle internal and external challenges, a critical path, and a driving force for achieving high-quality economic development and sustainable growth. Through extensive literature review and official documents, we found that China has not established a clear delineation for green economy industries. In contrast, there exists a more evident classification for digital economy industries in China. Therefore, based on existing official materials and scholars' research and utilizing China's IOT, we innovatively segment, adjust and classify green industries within the input-output framework. We then analyze the green economy and its industries. This constitutes the innovative aspect and theoretical significance of our study. Furthermore, through further literature review[2, 3], we found that our conclusions align with previous research, demonstrating the rationality and feasibility of our indicator selection.

1.	Yan X, Gong H, Wang B, Xie K. Countermeasures Analysis of Industrial Green Development in Xi'an City Based on WeaverThomas Model. Ecological Economy. 2017;33(12):72-6+118.

2.	Zhang Y, Wang W, Liang L, Wang D, Cui X, Wei W. Spatial-temporal pattern evolution and driving factors of China's energy efficiency under low-carbon economy. Science of the Total Environment. 2020;739:140197.

3.	Wang R. Ecological network analysis of China’s energy-related input from the supply side. Journal of Cleaner Production. 2020;272:122796.

**Comment 4:** The resolution of some graphs is low, especially in Figures 8 and 9, where the images appear blurred. Please adjust the resolution of the graphs according to the submission guidelines of this journal to ensure the clarity of the screen display. In addition, it is recommended that the text section be aligned at both ends.

**Response 4:** Thanks for your helpful comments. In our subsequent checking work, we found that the uploaded figures were compressed after the manuscript was produced as a PDF. In addition, we have re-processed all the Figures to be precise while having a resolution of 300 dpi. We put Figure 8 and Figure 9 below so that you can see clearly. Morever, we also set up the manuscript to be aligned at both ends.

Fig 8. Industry network community division map in 2012

Fig 9. Industry network community division map in 2020

**Comment 5:** The countermeasures proposed in the article are not ta rgeted enough. Please propose practical and instructive countermeasures based on the findings of the study.

**Response 5:** We highly appreciate the constructive suggestions of the reviewer regarding the Recommendations. We have revised this work in the Section 5.2: "1. Green and low-carbon industries need to strengthen their influence and solidify their position as the core strategic industries in China. It is not only a response to the optimization of China's industrial structure and the development trends of the world economy but also a requirement for China as a traditional agricultural country. "

"1. With the increasingly prominent role of the green economy in China's economic system, the status of China's green industry in the industrial structure is increasing. Therefore, it remains essential to focus on the long-term development of green industries. The green industry can increase its competitiveness and sustainable development capacity by increasing investment in green technology research and development and promoting the innovation and transformation of new energy and clean production technologies. At the same time, the government needs to formulate and improve laws and regulations to support green industries, ensure the implementation of environmental protection and energy conservation policies, and ensure their healthy development. In addition, it is also necessary to encourage enterprises and people to strengthen their awareness of environmental protection, and implement sustainable development strategies and green environmental protection concepts."

"2. As modern agriculture, industry, and services continue to develop, the adjustment and optimization of green industries are becoming increasingly important and demanding. Therefore, it is suggested to adopt a systematic approach. Strive to promote green industries' large-scale informatization and technological transformation, and lead traditional industries to rely on digital empowerment to reduce fees, reduce carbon emissions, and improve quality and efficiency." "2. With the development of modern agriculture, industry, and service industry, more and higher requirements are gradually being put forward for the adjustment and optimization of green industries. China is in the critical stage of developing the "321" industry, and the characteristics of multidisciplinary interdisciplinary and field integration are becoming increasingly prominent. Green industries can improve their competitive advantages and efficiency and accelerate their transformation by introducing advanced technologies and management models. It is crucial to integrate resources, technology, and markets, foster industrial synergies, and promote the integration and clustering of industries. At the same time, the government is guiding green industries to accelerate the establishment of innovation ecosystems and industrial clusters. It is also necessary to promote cooperation and innovation between different industries, realize the sharing and optimal allocation of resources, and enhance the relevance of industrial communities. "

"3. It is crucial to grasp the critical methodology when selecting key green industries for development. Priority should be given to industries with relative advantages, such as C4-electricity, heat production and supply, and A1-agricultural products. Overall green upgrading can be achieved by constructing and expanding their own industrial advantages. In particular, the electricity, heat production and supply industry requires China to optimize its energy structure further. This consideration is based not only on China being the world's largest agricultural and manufacturing country but also the world's largest energy producer. Effective promotion of new clean energy's manufacturing, storage, transportation, and consumption chain is necessary. It is also essential to increase the proportion of renewable energy in energy production and consumption while improving the ability to withstand significant energy risks and balancing the rational development of traditional energy sources." "3. The comprehensive index of the green industry indicates that the production and supply of agriculture, electricity, and heat significantly impact the overall economic network more than other industries. These areas are tangible priorities for China's green transition and economic development in the short term. Different policies and measures are needed for industries at different stages of development and trends. It is necessary for the leading industries to consolidate and coordinate the relationship and structure between the primary industries and the upstream and downstream related industries so that they can fully play their leading role. For the fast-growing service industry, while strengthening the mechanism construction of the industry itself, it is also necessary to pay attention to the integration of the digital economy industry and the green service industry and even the related development of other industries. At the same time, for green industries with great potential and growth space for future development, it is necessary to focus on development and promotion for a long time. Such policies should be given more preferential policies, guide them to accelerate the support of technological innovation and transfer, and introduce advanced green technology and management experience to promote the linkage effect of green industries to other industries. "
